# Horizontal bone augmentation and simultaneous implant placement using xenogeneic bone rings technique: a retrospective clinical study

**DOI:** 10.1038/s41598-021-84401-8

**Published:** 2021-03-02

**Authors:** Yude Ding, Lianfei Wang, Kuiwei Su, Jinxing Gao, Xiao Li, Gang Cheng

**Affiliations:** 1 Department of Stomatology, Zhejiang Provincial People’s Hospital, People’s Hospital of Hangzhou Medical College, Hangzhou, 310014 Zhejiang People’s Republic of China; 2 Department of Stomatology of Bengbu Medical College, Bengbu, Anhui People’s Republic of China

**Keywords:** Medical research, Outcomes research

## Abstract

This study evaluated the use of bone ring technique with xenogeneic bone grafts in treating horizontal alveolar bone defects. In total, 11 patients in need of horizontal bone augmentation treatment before implant placement were included in this retrospective study. All patients received simultaneous bone augmentation surgery and implant placement with xenogeneic bone ring grafts. We evaluated the postoperative efficacy of the bone ring technique with xenogeneic bone grafts using radiographical and clinical parameters. Survival rates of implants were 100%. Cone-beam computed tomography revealed that the xenogeneic bone ring graft had significantly sufficient horizontal bone augmentation below the implant neck platform to 0 mm, 1 mm, 2 mm, and 3 mm. It could also provide an excellent peri-implant tissue condition during the 1-year follow-up after loading. The bone ring technique with xenogeneic bone ring graft could increase and maintain horizontal bone mass in the region of the implant neck platforms in serious horizontal bone defects.

## Introduction

Over the years, implants have been utilized as the first-choice treatment to repair dental defects. Several factors critical to the long-term survival of implants and implant support for reconstruction have been identified. A prerequisite is a sufficient bone receptor site that allows for osseointegration on the surface of the implant especially in the esthetic area^1,2^.

Reports indicate that with implant placement, bone augmentation is often required before implantation in the site within 3 mm bone thickness or with a horizontal bone deficiency in the class III–IV defects^3,4^. Various surgical techniques have been described for augmenting the class III–IV horizontal defects, for example, guided bone regeneration (GBR) with titanium-mesh, bone block grafting, and distraction methods^5–7^. Although several clinical trials have proved that the above methods are effective in increasing bone volume^3,8^, they still have some drawbacks, among them, long treatment time for the two-staged implant placement, extra trauma in the secondary surgical site, and increased risk of infection^9–11^. In this context, increasing studies have explored simultaneous bone augmentation technique and implant placement (Bone Ring Technique) in recent years^6,12,13^.

Numerous studies have demonstrated that the bone ring technique integrated with an autogenous bone block can offer excellent outcome for dimensional bone augmentation^6,10,12–16^. With this technique, the implant is simultaneously placed with the bone augmentation procedure which both reduces the treatment time and maintains a sufficiently stable spatial structure^17^. Besides, due to the osteoinductive, osteoconductive, and osteogenic properties of autogenous grafts, clinicians in most cases graft the autogenous bone block to reconstruct the alveolar defects in this approach^18^. Nevertheless, autogenous bone harvesting usually requires an additional surgical site which may aggravate intraoperative pain and increase surgical time, complications, donor site morbidity^19^. Thus, inadequacies with the approach of autogenous bone ring deter patients from choosing this therapy. Also, it cannot provide sufficient bone grafts in cases where bone defects are severe in multiple implant areas.

Presently, a few clinical trials have started to explore the application of bone substitute materials in the bone ring technique to achieve sufficient bone augmentation and circumvent the deficiency of autogenous grafts^12,13,15^. A wealth of clinical trials demonstrated that the allogeneic grafts, xenografts, and synthetic materials are efficient just like the autogenous bone to increase bone volume thereby avoiding extra surgical site^3,8,20^. However, allogeneic grafts can easily fracture due to their poor biomechanical properties, also, they may induce immunologic tissue reactions and risk of disease transmission^21^. Therefore, xenogeneic bone substitute materials have been reported as an alternative to autologous bone grafts^22^. With the increasing research evidence, low manufacturing cost, the xenogeneic bone grafts are easy to acquire^23,24^. Moreover, researchers through animal experiments, have compared the histological characteristics of many widely used bone grafts for filling bone defects, such as autogenous bone, allogeneic grafts, bovine cancellous bone, calcium phosphate hydroxyapatite substitute, and calcium sulfate substitute. The highest histological score was reported using autogenous bone, followed by bovine cancellous bone, however, scores of all other grafts were inferior to bovine cancellous bone^25^.

Consequently, this study aimed to assess the clinical effect of xenogeneic grafts application following simultaneous implant placement with bone ring technique.

## Material and methods

### Initial situation

We performed a retrospective analysis on patients with class III–IV alveolar ridge defects who received treatment in the Department of Stomatology, Zhejiang Provincial People’s Hospital between December 2017 and June 2020. All patients had defects at the implant sites and were radiologically diagnosed with horizontal bone defects.All enrolled patients were fully informed of the study and they signed written informed consent. Notably, 11 patients were included in this study after meeting the following inclusion criteria:

1. When the three-dimensional bone defects in the missing teeth area should be classified as class III-IV defects in the horizontal direction.
2. Patients with good general health without any prolonged disorders, such as TMJ disorders and any other systemic disease.
3. Patients who cooperated with the preoperative examination and postoperative follow-up with subjective planting intention.

Besides, patients with conditions of drug or smoke abuse, uncontrolled periodontitis, those who underwent bisphosphonate and irradiation to the head and neck region within 5 years were excluded from the study.

### Ethics

The institutional ethics committee of Zhejiang Provincial People’s Hospital approved the study (No. 2017KY049), which was performed according to the principles of the Declaration of Helsinki. Written informed consent was obtained from the patients or from the guardians of the patients younger than 18 years. All identifying images released in this article were authorized for publication by the patient and his guardians.

### Surgical procedures

**Figure 1:**
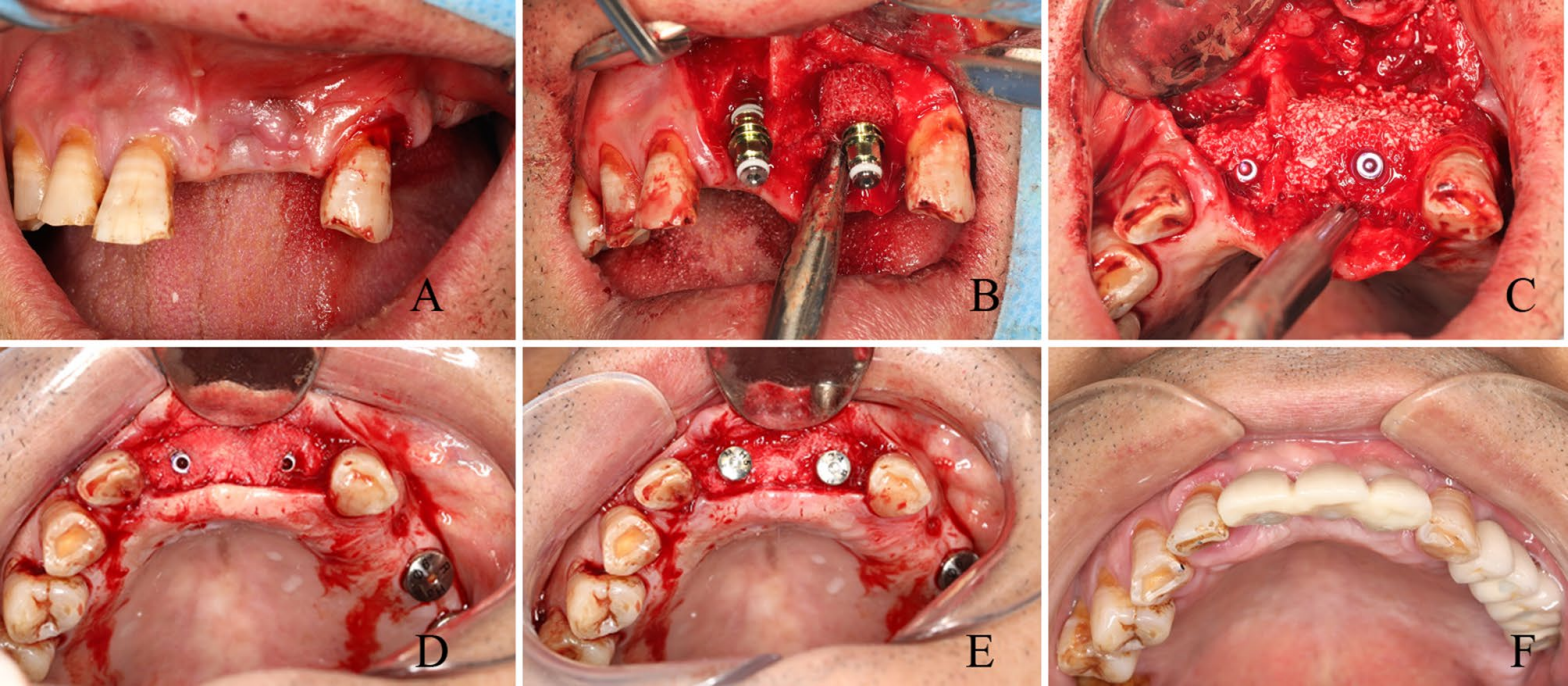
(**A**) A preoperation picture showing tooth loss in a front view; (**B**) the implant was inserted through the xenogeneic bone ring into the alveolar bone; (**C**) bovine bone substitute filled the remaining free space in the defect; (**D**) re-entry was performed with a full-thickness mucoperiosteum flap after 6 months; (**E**) healing abutments were placed; (**F**) crowns were rehabilitated 8 months later after the surgery.

All selected patients underwent periodontal and dental examinations before surgery and received effective periodontal treatment. One experienced surgeon conducted the surgeries with local anesthesia (Articaine hydrochloride 4%; Epinephrine, 1:100,000). First, the implant site was prepared using the pioneer drill of the implant system with midcrestal incision and buccal mucoperiosteal flap. Then, the bone defective area around the implant site was prepared using the trephine bur to fit the recipient area to the xenogeneic bone ring graft. This was followed by insertion of the dental implant through the xenogeneic bone ring which was in 7 mm outer ring diameter and the implant was positioned about 1–2 mm below the coronal border of the ring. Thereafter, the graft was fixated with the dental implant, about 3–4 mm of the implant length was into the bone ring, and more than 5 mm into the alveolar bone. The implants were provided with primary stability using an insertion torque of 25–35 Ncm. The remaining free space in the defect was filled with bovine bone substitute (Geistlich Bio‐oss, Geistlich Pharma, Wolhusen, Switzerland) with the patient's blood and covered with a collagen membrane (Geistlich Bio‐Gide, Geistlich Pharma, Wolhusen, Switzerland). Eventually, the flaps were repositioned and sutured free of tension carefully to prevent dehiscence (Fig. 1). All Patients were administered with 500 mg amoxicillin thrice a day for 3 days.

### Prosthetic procedure

The implants were submerged for 6 months of healing. Then, re-entry was conducted with a full-thickness mucoperiosteum flap at the same surgical site of bone augmentation and healing abutments were placed for 2 months to shape the gingiva, whereas the crowns were rehabilitated 8 months post-surgery.

### Clinical evaluation

**Figure 2:**
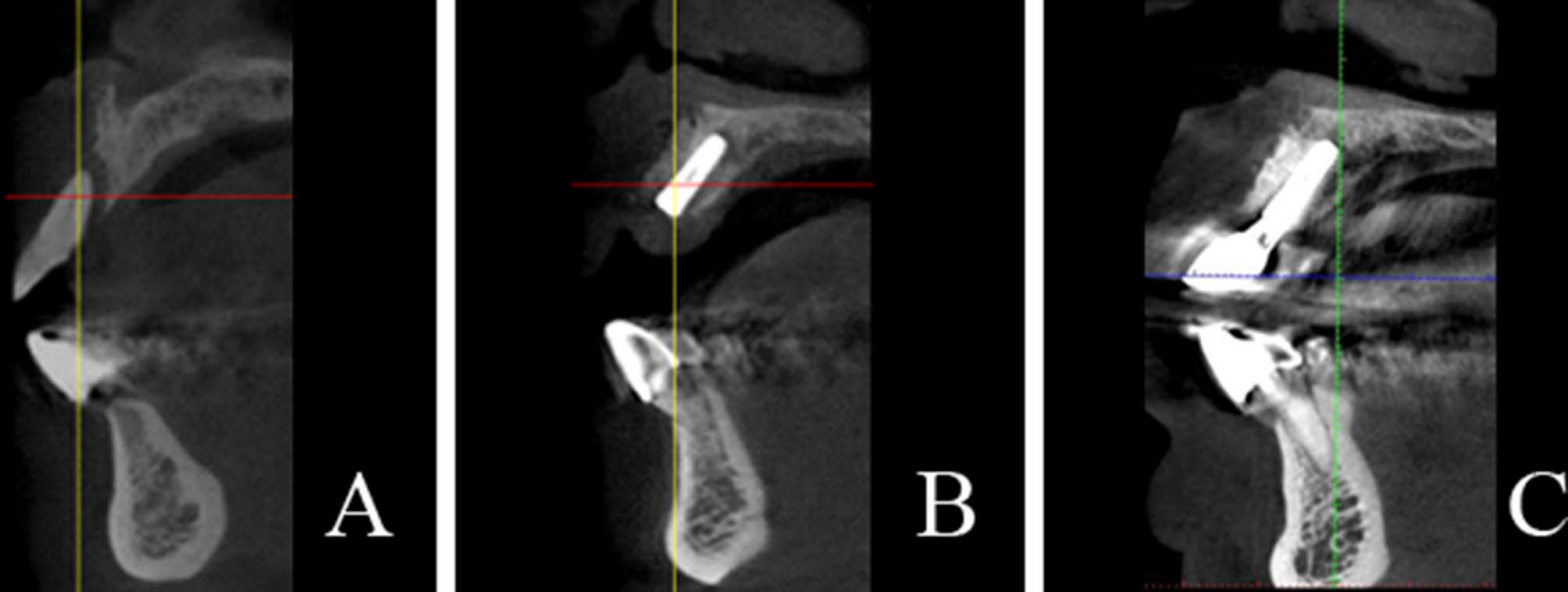
(**A**) Cone-beam computed tomography (CBCT) pictures showing bone defect preoperation; (**B**) bone profile with an implant and xenogeneic bone ring after 6 months after the surgery; (**C**) bone profile with implant 1 year after loading.

**Figure 3:**
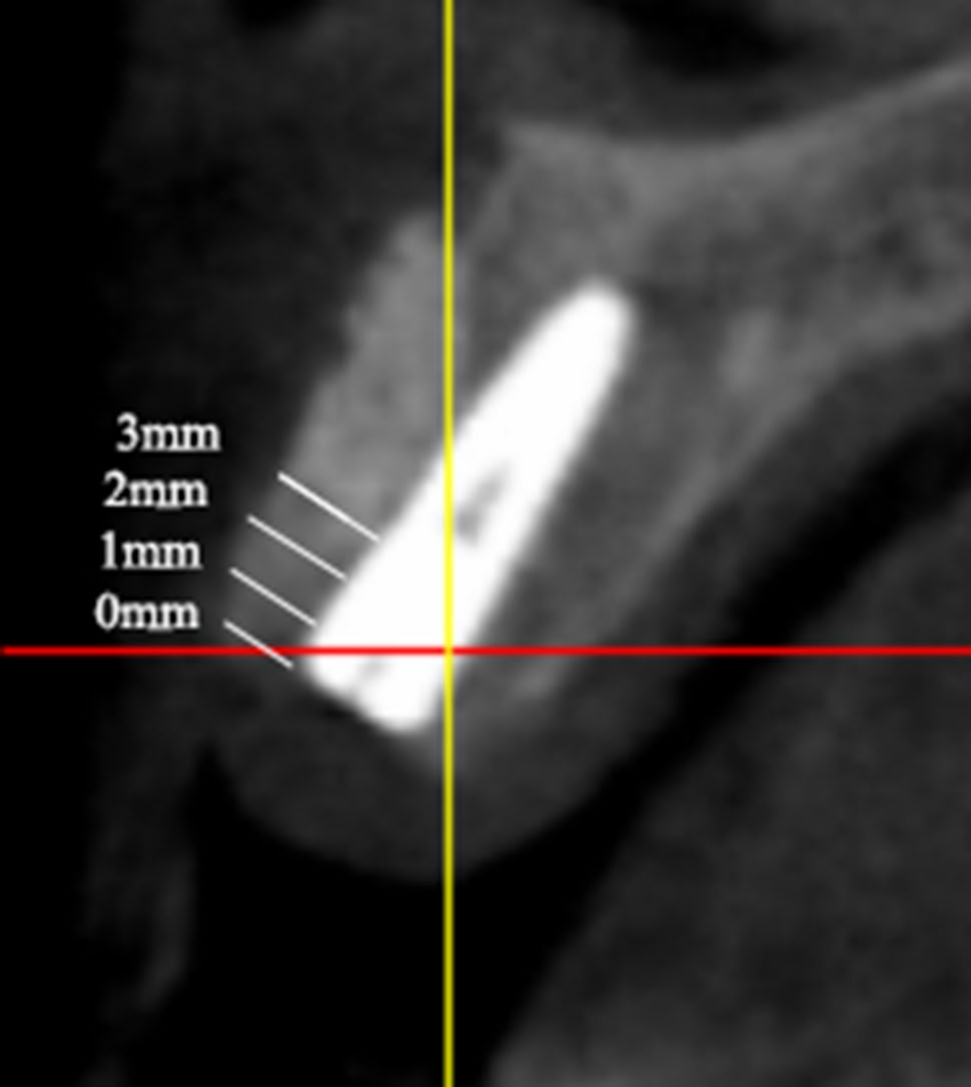
Measurement of bone widths at all four different levels (below the implant neck platform to 0 mm, 1 mm, 2 mm, and 3 mm).

Radiographic examinations were performed at preoperation, immediate postoperative, and 12 months after prosthetic restorations using cone-beam computed tomography (CBCT) (Fig. 2). Thus, we could evaluate the effects of bone augmentation and the progression of bone resorption especially at the implant shoulders and the apical zones of the implants. A measurement of the bone widths was taken around the implants in the plane perpendicular to the long axis of the implants by utilizing a CBCT ProMax 3D Mid unit (Planmeca, Helsinki, Finlandia). We took measurements in the parallel planes which were below the implant neck platform to 0 mm, 1 mm, 2 mm, and 3 mm (Fig. 3). Therefore, the amount of horizontal bone mass could be obtained in each part to evaluate the horizontal bone gain and the resorption of the xenogeneic bone. During the process, 2 physicians repeated the measurement in triplicate and re-measured it 2 weeks later. The average data of the repetitions were taken for statistical analysis.

Furthermore, we evaluated other clinical parameters of the implants to assess the treatment outcomes of this study in terms of implant success rates, peri-implant tissue condition (including plaque index (PI), bleeding on probing (BOP) and gingival index (GI)) and complications during the 1-year follow-up period after loading.

- PI = score range of 0–3: no plaque (score = 0), a film of plaque cannot be seen with the naked eye but only by using a probe(score = 1), moderate accumulation of deposits (score = 2) and abundance of soft matter (score = 3)^26^.
- BOP = score range of 0–3: no bleeding (score = 0), small punctuated bleeding (score = 1), redline bleeding on the margin(score = 2) or profuse bleeding (score = 3)^26^.
- GI = score range of 0–3: no (score = 0), mild (score = 1), moderate(score = 2) or severe (score = 3) inflammation^27^.

### Statistical analyses

The statistical analysis was performed using SPSS 19.0 (IBM Corp., Armonk, NY, USA) (https://www.hearne.software/spss-selection). The same process was repeated 1 week later to assess measurement accuracy.Descriptive data were presented as means ± SDs. Descriptive data of different follow-up periods were examined using the Student t-test, p-value < 0.05 was considered statistically significant.

## Results

We included 11 patients (8 males and 3 females with an average age of 49.91 ± 12.95 years) at the time of implant placement. Detailed information of all patients is highlighted in Table 1. During the follow-up period, one patient presented with a fracture of the wound 1-week post-surgery but healed after receiving secondary intervention without further developing complications. Twelve implants were performed in both anterior and posterior regions. Notably, the implant success rate was 100%, following the commonly accepted implant success criteria (the ICOI Pisa consensus in 2007)^28,29^. No major biological complications were witnessed. The average values of PI, BOP, and GI index of the implants were less than two points at the immediate prosthetic restorations (T1’), and 1 year after loading (T2), this indicated the stability of peri‐implant tissue condition. However, no significant difference was observed during the observation period (Table 2). And we found the marginal bone loss was 1.46 ± 0.38 mm in the buccal side of the implants through the CBCT 1 year after prosthetic restorations.

**Table 1:** Characteristics of the study patients.

Number	Sex	Age (years)	Reason for defect	Location of region site
1	Male	56	Tooth loss owing to periodontal disease	16
2	Male	57	Long-term absence of teeth	26
3	Female	53	Tooth loss owing to periodontal disease	21
4	Male	61	Tooth loss owing to periodontal disease	22
5	Female	20	Tooth loss owing to infection	44
6	Male	58	Tooth loss owing to infection	34
7	Male	63	Long-term absensce of teeth	44
8	Male	42	Tooth loss owing to infection	26, 27
9	Female	32	Tooth loss owing to infection	36
10	Male	47	Tooth loss owing to periodontal disease	21
11	Male	60	Tooth loss owing to periodontal disease	46

**Table 2:** The scores of the peri-implant tissue condition.

	T1'	T2	*P*
PI	0.83 ± 0.69	1.17 ± 0.55	0.104
BOP	1.17 ± 0.69	1.33 ± 0.75	0.586
GI	1.33 ± 0.47	1.58 ± 0.49	0.191

T1': Immediate prosthetic restorations; T2: 12 months after prosthetic restorations.

For horizontal bone gain, the mean bone gain at four planes (0 mm, 1 mm, 2 mm, 3 mm) were 2.40 ± 0.31 mm, 2.81 ± 0.45 mm, 3.35 ± 0.50 mm, 3.76 ± 0.53 mm at immediate postoperative and 1.95 ± 0.19 mm, 2.39 ± 0.38 mm, 2.91 ± 0.56 mm, 3.28 ± 0.63 mm at 12 months after prosthetic restorations. Notably, significant decreases which indicated the mean bone resorption were observed at four planes (p < 0.05). The mean bone resorption at four planes were 0.45 ± 0.28 mm, 0.41 ± 0.36 mm, 0.43 ± 0.31 mm, 0.48 ± 0.31 mm (Table 3), and bone absorption rates were 17.78 ± 9.03%, 13.86 ± 10.55%, 13.02 ± 8.85%, 12.96 ± 8.36% at four levels.

**Table 3:** The bone gain and resorption of the the xenogeneic bone ring around the implants in the study.

Different planes	T1 (mm)	T2 (mm)	T2–T1 (mm)	*t*	*p*
0	2.40 ± 0.31	1.95 ± 0.19	− 0.45 ± 0.28	5.3	0.00*
1	2.81 ± 0.45	2.39 ± 0.38	− 0.41 ± 0.36	3.83	0.00*
2	3.35 ± 0.50	2.91 ± 0.56	− 0.43 ± 0.31	4.56	0.00*
3	3.76 ± 0.53	3.28 ± 0.63	− 0.48 ± 0.31	5.08	0.00*

Different planes: different parallel planes below the implant neck platform (mm); T1: Immediate postoperative; T2:12 months after prosthetic restorations.

p*: p < 0.01.

## Discussion

The bone ring technique, first reported by Giesenhagen, can offer three‑dimensional augmentation with simultaneous implant placement in a single-stage procedure^30–32^. Bone ring technique is highly significant compared to conventional bone grafting in treating serious defects, in terms of reducing the whole treatment time^31–34^. However, the autogenous bone ring technique may provide an extra surgical site, increase the risk of infection, and paraesthesia of chin through the incisal nerve injury^19^. All the attributed complications may discourage patients from going for this treatment. Thus, the application of bone substitute materials is regarded to be increasingly vital and urgent for bone ring technique.

Based on previous reports, the allogeneic bone ring grafts had been used in a few clinical trials^12,13,15,35^ and got sufficient bone augmentation. Still, the application of allogeneic grafts is restricted by factors such as high cost, limited sources, the risk of disease transmission, and immunologic tissue reactions. In this context, the xenogeneic bone has been widely used clinically for its excellent biomechanical properties, bone conductibilities, and a wide range of sources. A study revealed that bovine cancellous bone exhibits better histological characteristics than other bone substitutes^25^. Therefore, we chose the bovine cancellous bone (Hell-all, Zhenghai Biotechnology Co., Ltd, Yantai, China) as the bone ring material to repair serious dental defects. The material is made of bovine cancellous bone after decellularization and degreasing treatment, thereby fully retains the type I collagen and hydroxyapatite components in the natural bone composition. The natural three-dimensional porous structure of the material (aperture 50–600 μm) can promote new bone growth and regulate bone regeneration^36^.

Bone cylinders made from bovine cancellous were utilized for the grafts, and we used the pioneer drill of the implant system to change the bone substitute to the ring shape. The bone ring and the implant site were drilled via the continued-drills of the implant to ensure a high degree of match between the implant and the graft. Since the implant diameter needed to be more than 3 mm for strength, at least 1–2 mm space was reserved around the shoulder of the implant to ensure the success of bone grafting. The application of the xenogeneic bone ring technique requires that the medial distal dimension of the receptor site be more than 7 mm. In this study, we found that the wide and flat platform of the bone ring made it difficult to obtain perfect gingival nipples for single implant restoration in the aesthetic area of the anterior teeth. Therefore, we chose the 7 mm diameter xenogeneic bone cylinder as the graft measure and used the bone ring in the posterior region and the region of multiple anterior teeth loss. For the above reasons, it is unsuitable to use the xenogeneic bone ring to repair the defect in a narrow mesiodistal dimension site, particularly, in the aesthetic area.

According to previous investigations, the new bone formation ratios of deproteinized bovine bone (DPB) and human-derived allogenic bone (HALG) have been revealed to be about 43.63 ± 6.30%, 45.25 ± 6.71% using the titanium barriers in severe defects^37,38^. There was no significant difference in the rate of new bone formation between the two materials. Therefore, we selected the xenogeneic bone grafts which could be easy to acquire as the material to assess the clinical effect of xenogeneic grafts application following simultaneous implant placement with bone ring technique in horizontal bone defect implantations. Herein, we showed that the xenogeneic bone ring had extremely efficient abilities in maintaining a stable space, especially in the region of the implant neck platform. The new bone formation ratios around the neck of the implants ranged from 82.22 ± 9.03 to 87.04 ± 8.36%, and bone width augmentation was revealed to be more than 1.5 mm after 12 months of prosthetic restorations. The great graft stability and mechanical support of the xenogeneic bone ring technique could obtain satisfactory bone augmentation to repair the tooth loss with severe bone defects in a horizontal direction.

In recent study, Yohei reported that osseointegration in the vertical bone augmented area showed low new bone to implant contact in the xenogeneic group^39^. While they also pointed out due to the surrounding of collagen fibers, osteoblasts could not grow into the xenogeneic ring structure. It might be the reason why xenogeneic ring presented low new bone formation in the animal experiment. They thought covering with a membrane was necessary to prevent tissue ingrowth other than bone when using xenogeneic bone material for vertical bone augmentation. Therefore,we mainly applied the xenogeneic bone rings technique to the cases of horizontal bone defects, because we believe that in the cases of vertical bone defects, the xenobone ring technique because of the existence of the soft tissue pressure may not be able to obtain satisfactory results. The xenogeneic bone ring technique is not suitable for complete vertical bone defects, such as class V–VI defects. So In this study, we studied the clinical application effect of xenogenic bone ring technique in horizontal bone defect implantation, and reduced the influence of surrounding collagen fibers through membrane coverage.

Of course, further researches are needed to evaluate the incidence of complications and the marginal bone loss after a longer observational time and a larger-scale clinical trial to validate this approach. Of note, one of the keys to utilizing this method is to control the position of the ring in relation to the implant position when preparing for implantation. Therefore, future research directions should be geared towards integrating bone ring technology with digital technology to control the precision of the implantation process.

## Conclusion

In summary, we affirmed that the bone ring technique with xenogeneic bone ring graft is a predictable treatment option for future horizontal bone augmentation. Our study illustrated the benefits of the xenogeneic bone ring for the regeneration of alveolar bone. This approach could increase and maintain horizontal bone mass in class III–IV defects in the horizontal direction. It could also reduce the treatment time with simultaneous implant placement in a single‑stage procedure, and avoid additional surgical site. Therefore, this technique may be a favorable treatment choice regarding severe horizontally bone defects.
